# Transcriptomic analysis reveals the mechanism underlying salinity-induced morphological changes in *Skeletonema subsalsum*

**DOI:** 10.3389/fmicb.2024.1476738

**Published:** 2024-10-29

**Authors:** Jingwen Hu, Ya Zheng, Shuang Yang, Lin Yang, Qingmin You, Quanxi Wang

**Affiliations:** ^1^Laboratory of Algae and Environment, College of Life Sciences, Shanghai Normal University, Shanghai, China; ^2^Laboratory of Environmental Ecology and Engineering, College of Life Sciences, Hengshui University, Hengshui, China

**Keywords:** diatoms, salinity, morphological changes, cell wall morphogenesis, molecular mechanism

## Abstract

Diatom cell walls are diverse and unique, providing the basis for species identification and supporting the ecological and economic value of diatoms. However, these important structures sometimes change in response to environmental fluctuations, especially under salt adaptation. Although studies have shown that salinity induces morphological plasticity changes in diatom cell walls, most research has focused on physiological responses rather than molecular mechanisms. In this study, *Skeletonema subsalsum* was cultured under four salinity conditions (0, 3, 6, 12). Through morphological and physiological methods, we found that salinity increased the cell diameter, protrusion lengths, distance between adjacent cells (DBCs), and nanopore size, while reducing cell height and silicification degree. To further investigate the mechanism underlying morphological changes in *S. subsalsum*, complementary transcriptome analysis was performed. In total, 20,138 differentially expressed genes (DEGs) were identified among the four treatments. Among them, 231 DEGs were screened and found to be closely associated with morphological changes, of which 107 were downregulated and 124 were upregulated. The findings demonstrated that elevated salinity inhibited silicon transport and deposition via downregulating the expression of DEGs involved in functions including chitin metabolism, putrescine metabolism, and vesicle transport, resulting in reduced silicon content and cell height. Increased salinity promoted the expression of DEGs related to microtubules (MTs), actin, and ubiquitin, which synchronously induced morphological changes. These findings provide a more comprehensive understanding of the salt tolerance of algae and a foundation for future studies on cell wall morphogenesis.

## 1 Introduction

Diatoms, the unicellular and photoautotrophic organisms, are among the most ecologically successful microalgae (Cohen et al., 2018). Diatoms contribute at least 20% of global primary production (Creveld et al., 2016) and carbon dioxide fixation (Gügi et al., 2015), making them critical for regulating the global climate. In addition, diatoms are central to the biogeochemical cycling of elements such as nitrogen, silicon, and iron (Zhao et al., 2022). Uniquely, diatoms have delicate siliceous cell walls, also called frustules. These structures not only channel and focus light (Stefano et al., 2007), which may help to improve the photosynthetic efficiency of diatoms (Parker and Townley, 2007), but also provide mechanical protection from adverse environmental stress (Hamm et al., 2003), which may account for the ecological success of diatoms and endow diatoms with even greater ecological value (Skeffington et al., 2022). Moreover, frustules of diatoms exhibit diverse structures from the nanoscale to millimeter scale, which are instructive for constructing photonic structures, chemo/biosensing and new nano-devices (Gordon et al., 2009).

The frustule structures of diatoms are relatively stable, and their shapes and patterns are considered an important basis for the identification of diatoms. However, the frustules of some diatoms can change with environmental fluctuations. To date, the influences of various environmental factors, comprising temperature, salinity, pH, heavy metals, light, nutrient concentrations, and biotic factors, on the cell wall morphology of diatoms have been investigated (Su et al., 2018). Among these factors, the influence of salinity has received the most attention. The effects of salinity on the cell size and fibula density have been found in *Nitzschia* (Trobajo et al., 2004) and *Skeletonema* (Balzano et al., 2011). In addition, changes in the cell shape, protrusion length, and protrusion number with salinity were reported in *Cyclotella* (Hakansson and Chepurnov, 2011). Aizdaicher and Markina (2010) demonstrated that *Corethron hystrix* grown at lower salinities had a taller stature and shorter spines. Kamakura et al. (2022) observed the changes of valve face and ocelli in *Pleurosira laevis* in response to salinity. Moreover, Vrieling et al. (2007) and Leterme et al. (2010) have reported that greater salinity increases the size of nanopores in *Thalassiosira punctigera*, *Thalassiosira weissflogii*, *Nitzschia salinarum* and *Cocconeis placentula*. Although the morphological plasticity of diatoms in response to salinity has been widely recognized, little attention has been paid to the underlying mechanism driving these morphological changes.

*Skeletonema subsalsum*, a typical chain-forming diatom, is an euryhaline planktonic alga (Hasle and Evensen, 1975) that inhabits waters from rivers to coastlines. We previously noted that increased salinity caused changes in the cell outline, fultoportula processes (FPPs) lengths, and distance between adjacent cells (DBCs) of *S. subsalsum* (Hu et al., 2024). *S. subsalsum* is regarded as an ideal material to explore the mechanism of morphological changes under salinity due to its extensive salinity adaptability and morphological plasticity. Additionally, morphological changes in diatoms are closely associated with cell wall morphogenesis. Here, it is hypothesized that salinity affects cell wall morphogenesis and thus induces morphological changes. Therefore, exploring the mechanism of morphological changes under salinity adaptation is also a new direction for the study of cell wall morphogenesis.

In this study, *S. subsalsum* was selected as the experimental material and cultured under four salinity levels. Integrating morphology, physiology, transcriptomics, and quantitative real-time polymerase chain reaction (qRT-PCR) approaches, we described the growth and metabolic expression patterns of *S. subsalsum*. Differentially expressed genes (DEGs) related to cell wall formation were screened out and analyzed, aiming to elucidate the molecular mechanism of morphological changes in *S. subsalsum*, to provide more perspectives regarding the mechanism of cell wall development. The findings of this study will help researchers gain a thorough insight into how diatoms respond to salinity, offer a foundation for future research on cell wall morphogenesis in diatoms and provide more opportunities to search for new synthesis methods of nanostructured silica materials.

## 2 Materials and methods

### 2.1 Plant materials and salt treatment

*S. subsalsum* strains were collected from a river in Shanghai (China) and isolated by capillary pipettes under a Nikon Ts2 inverted microscope. The isolated strain was cultivated in CSI medium (Kasai et al., 2004) with four salinity levels (0, 3, 6, 12). Cultures were kept at 24°C under a 12 h꞉12 h light/dark photoperiod at 62.5 μmol photons/(m^2^·s). We conducted a semi-continuous batch culture and transferred the cultures into fresh media every 4 days.

### 2.2 Morphological observations and measurements

Four salinity samples, namely CK (salinity = 0, S0), lower salinity (salinity = 3, LS), moderate salinity (salinity = 6, MS), and higher salinity (salinity = 12, HS), were observed under an Eclipse 80i microscope (Nikon, Tokyo, Japan) and photographed with a digital camera (DS-Ri2, Nikon, Tokyo, Japan). Cell height and frustule diameter (cell width) were measured by Image J software. Fifty such measurements were performed on randomly selected cells at each salinity level. One-way ANOVA was used for difference analysis.

Four samples were treated with concentrated nitric acid using the Microwave Accelerated Reaction System (Model MARS, CEM Corporation, Charlotte, United States) (Parr et al., 2004; Yu et al., 2017). Samples were washed about seven times and kept in 95% ethanol. Cleaned diatom frustules were air-dried onto cover slips and attached to copper stubs, coated with ~15-nm gold palladium using a sputter coater (Hitachi E-1045) (Hu et al., 2024). Diatoms were examined using a Hitachi SU 8010 SEM (2 kV) and its images were compiled with Adobe Photoshop CS4 Extended (Hu et al., 2023). Four parameters, namely FPPs lengths, rimoportulae processes (RPPs) lengths, DBCs, and nanopore size, were also measured using Image J software. The RPPs lengths of thirty randomly selected valves were measured. The DBCs of fifty randomly selected valves were detected. In measurements of FPPs lengths and nanopore size, three measurements were carried out on each cell and a “cell mean” was calculated, then fifty “cell mean” were used to compute the mean at each salinity. One-way ANOVA was used for difference analysis.

### 2.3 Physiological parameter analysis

The cell density was measured daily by the blood cell counting plate method (Absher, 1973). The silicon content in the culture was measured using inductively coupled plasma optical emission spectrometry. Differences were analyzed using *t*-tests to determine significance. Results are visualized by GraphPad Prism (version 9.4.0).

### 2.4 Transcriptomic analysis

#### 2.4.1 Sample collection and RNA preparation

After 4 days culture, *S. subsalsum* cells were harvested by centrifugation at 6,000 × g for 8 min. The total RNA was extracted using TRIZOL reagent (Vazyme, Nanjing, China) following manufacturer’s recommendations (Zhang et al., 2016). Three biological replicates were performed per treatment. RNA degradation and contamination were monitored on 1% agarose gels. Purity of RNA was determined with Nano Photometer^®^ spectrophotometer (IMPLEN, CA, United States). RNA concentration was assessed by Qubit^®^ RNA Assay Kit in Qubit^®^2.0 Flurometer (Life Technologies, CA, United States). RNA integrity was monitored using the RNA Nano 6000 Assay Kit of the Agilent Bioanalyzer 2100 system (Agilent Technologies, CA, United States).

#### 2.4.2 Library construction and sequencing

A total of 2 μg RNA per sample was used as input material for RNA sample preparation. Sequencing libraries were constructed using the VAHTSTM mRNA-seq V2 Library Prep Kit for Illumina^®^ according to the manufacturer’s instructions and index codes were added to each sample. mRNA purification was conducted using poly-T oligo-attached magnetic beads (Yuan et al., 2024). The obtained RNA was fragmented with VAHTSTM First Strand Synthesis Reaction Buffer (5X). cDNA was synthesized using random hexamer primer, M-MuLV Reverse Transcriptase (RNase H-) and DNA polymerase I (Zhang et al., 2014). After adenylation of 3′ ends of DNA fragments, adaptor was ligated to prepare for library. The library fragments were purified with AMPure XP system to select cDNA fragments of preferentially 150–200 bp in length (Beckman Coulter, Beverly, United States). PCR was performed with universal PCR primers and index (X) primer. PCR products were purified and library quality was assessed on the Agilent Bioanalyzer 2100 system. Paired-end sequencing of the library was performed on the HiSeq XTen sequencers (Illumina, San Diego, CA).

#### 2.4.3 *De novo* assembly and gene annotation

FastQC (version 0.11.2) was used for evaluating the quality of sequenced results. Raw data were trimmed using TGICL (version 2.1) to remove the sequences with N bases, low-quality reads (Q < 20) and short reads (<35 nt in length). A sliding window method was used to remove the base value less than 20 of reads tail (Dang et al., 2013). The remaining clean data was *de novo* assembled into transcripts using Trinity (version 2.8.5) (Eisen et al., 1998). Transcripts were clustered to minimize redundancy, and the longest sequence in each cluster was preserved and designated as a unigene.

Six public databases, including NCBI non-redundant protein sequences (NR), Gene Ontology (GO), Kyoto Encyclopedia of Genes and Genomes (KEGG), Clusters of Orthologous Groups of proteins (COG), Pfma, SwissProt, were used to annotate the unigenes with a threshold *e*-value < 1e^−5^ as in Zhang et al. (2014). The reference species and annotation results from these databases were compared to determine biological functions and metabolic pathways of each unigene.

#### 2.4.4 Analysis of DEGs

Quantified analysis was performed by RSEM (version 1.3.1). DESeq2 (version 1.24.0) was employed to calculate the Fold Change (FC) of unigenes. DEGs were defined with the criteria of |log_2_FC| ≥ 1 and *P* adjust < 0.05.

Automated meta-analysis tools are provided in Metascape. Multiple data sources such as GO and KEGG are integrated to complete pathway enrichment and biological processes notes (Cohen et al., 2018). Goatools software was used to carry out GO/KEGG enrichment analysis of DEGs (Tang et al., 2015). Protein–protein interaction network was performed with R igraph package.

### 2.5 qRT-PCR validation

Total RNA of each sample was extracted for qRT-PCR analysis as described above. First strand cDNA was converted using a HiScript^®^ III 1st Strand cDNA Synthesis Kit (+gDNA wiper) (Vazyme, Nanjing, China). qRT-PCR was performed in triplicate on a Light Cycler 480 II System (Roche, Shanghai, China) using Cham Q Universal SYBR qPCR Master Mix (Vazyme, Nanjing, China). The expression levels of tubulin alpha (*tubA*), tubulin beta (*tubB*), chitin synthase (*CHS*), chitinase (*CHI*), 1,3-beta-D-glucan synthase (*CalS*), glutamine-fructose-6-phosphate aminotransferase (*GFPT*), glutamine synthetase (*GS*), and *SecY* were examined. Primers were designed by Primer primer 5 software. All primers used in this study are listed in Supplementary Table S1. The relative expression levels of selected DEGs were normalized to nuclear encoded small subunit of rDNA (nSSU) according to the 2^−ΔΔCt^ method (Li et al., 2020). One-way ANOVA was used to compare the differences between the CK and treatment groups. Box plots were constructed with GraphPad Prism 9 software.

## 3 Results

### 3.1 Morphology changes of *Skeletonema subsalsum* at various salt concentrations

**Figure 1:**
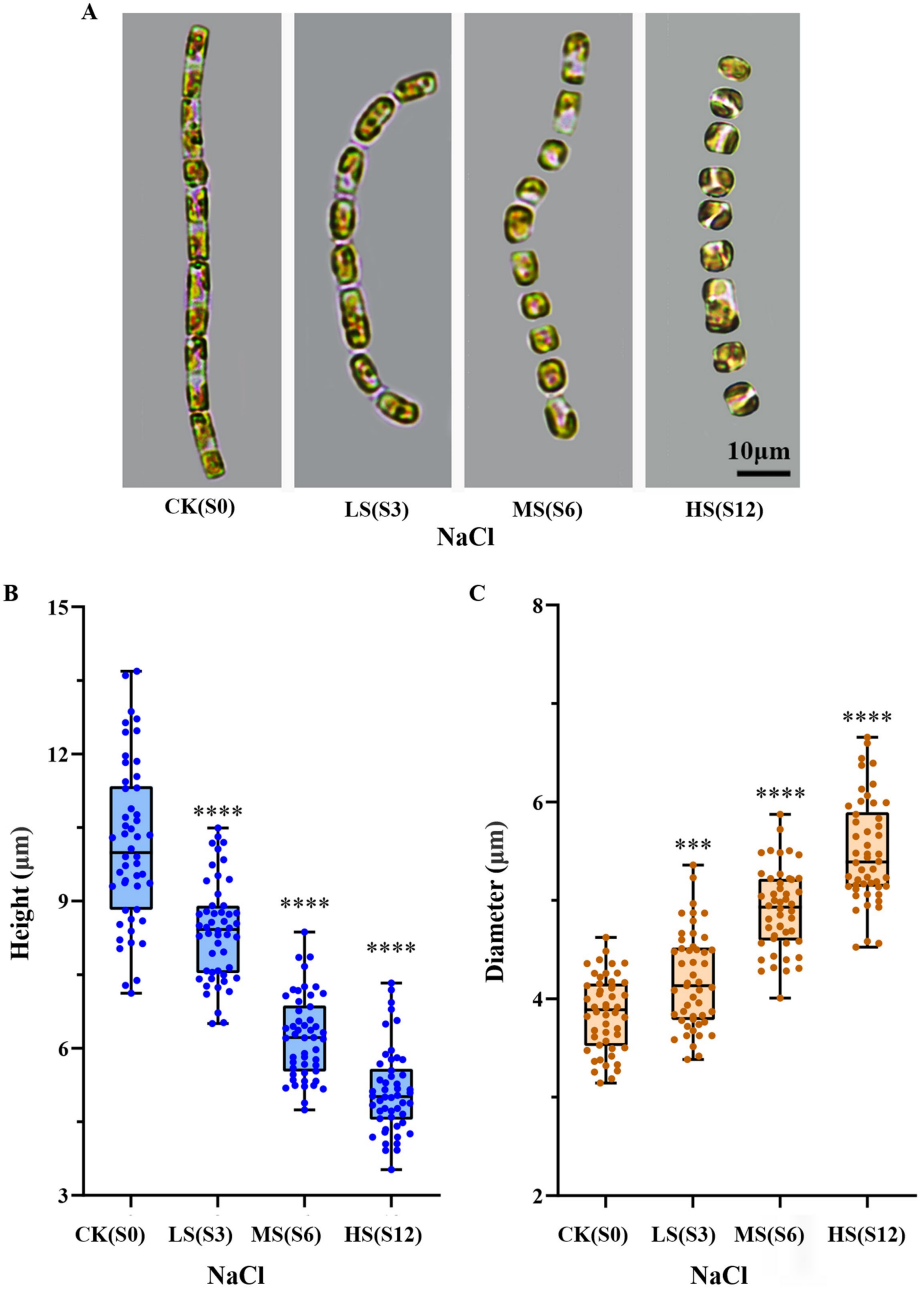
Changes in the shape and size of *Skeletonema subsalsum* with increasing salinity. (A) Morphological changes observed under the LM. (B) Quantitative analyses (box plots) of the cell height across varying salinity levels (*n* = 50). (C) Quantitative analyses (box plots) of the cell diameter at various salinity levels (*n* = 50). In (B,C), whisker ends indicate upper and lower quartile and the median is the horizontal line in the box. Each circle represents a measurement of height or diameter. One-way ANOVA was used for difference analysis, *** and **** indicate significant differences between treatment groups and CK(S0) are *p* < 0.001 and *p* < 0.0001, respectively.

*S. subsalsum* can grow within the salinity range of 0–12. A pronounced influence of salinity on the cell morphology of *S. subsalsum* was revealed under LM (Figure 1A) and verified by measurements conducted on photographed cells (Figures 1B,C). When the cells were grown at LS, their valve faces remained flat. There was limited variation in cell height (10.16–8.39 μm) and frustule diameter (3.86–4.21 μm). At MS, the morphological changes of cells were relatively obvious, with the mean height reduced to 6.81 μm and the diameter increased to 4.90 μm. Notably, the frustules began to become dome-shaped. At HS, the cell height (5.12 μm) and diameter (5.39 μm) continued to change, and eventually the cells became completely round or oval.

**Figure 2:**
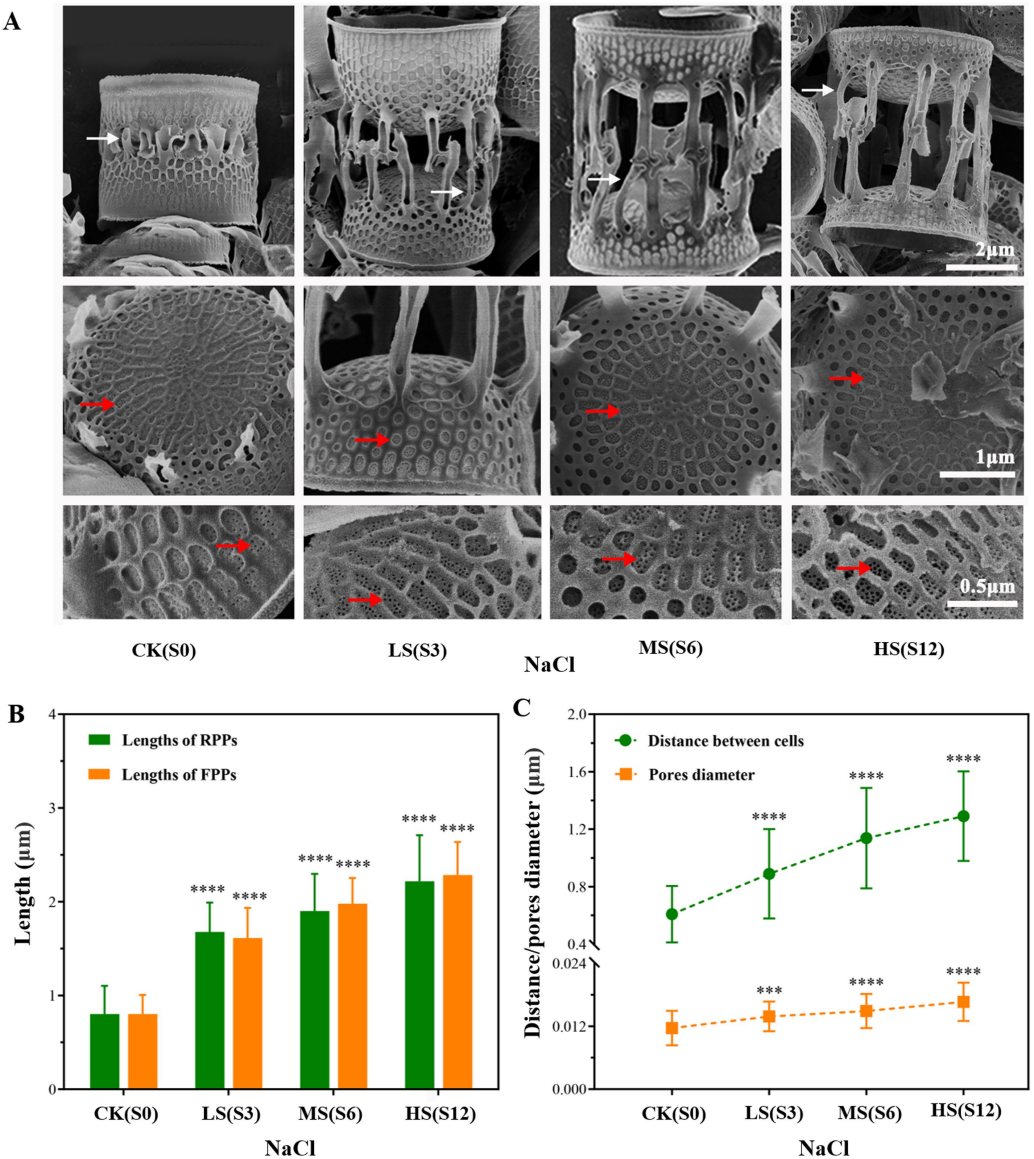
Electron micrographs of cleaned frustules of *Skeletonema subsalsum* under different salinities. (A) Morphological changes observed under a scanning electron microscope; white arrowheads point to rimoportulae processes and red arrowheads show nanopores. (B) Bar chart displaying the lengths of fultoportula processes (*n* = 50) and rimoportulae processes (*n* = 30). (C) Line chart displaying the nanopore size (*n* = 50) and distance between adjacent cells (*n* = 50) of *Skeletonema subsalsum* under different salinities. In (B,C), the error bars represent the strand deviations of measured height and diameter. One-way ANOVA was used for difference analysis, *** and **** indicate significant differences between treatment groups and CK(S0) are *p* < 0.001 and *p* < 0.0001, respectively.

To obtain a deeper understanding of the morphological changes in cells, SEM was employed to observe and measure the DBCs, the lengths of FPPs and RPPs, and the size of nanopores in samples subjected to four salinity levels (Figure 2A). The results showed that the four parameters changed synergistically, each increasing with higher salinity levels. The lengths of FPPs and RPPs varied greatly with salinity, ranging from 0.80 to 2.45 μm and from 0.80 to 2.22 μm, respectively (Figure 2B). The DBCs increased to 1.29 μm, and the nanopore size enlarged by 0.005 μm (Figure 2C).

### 3.2 Physiological responses of *Skeletonema subsalsum* to salinity

**Figure 3:**
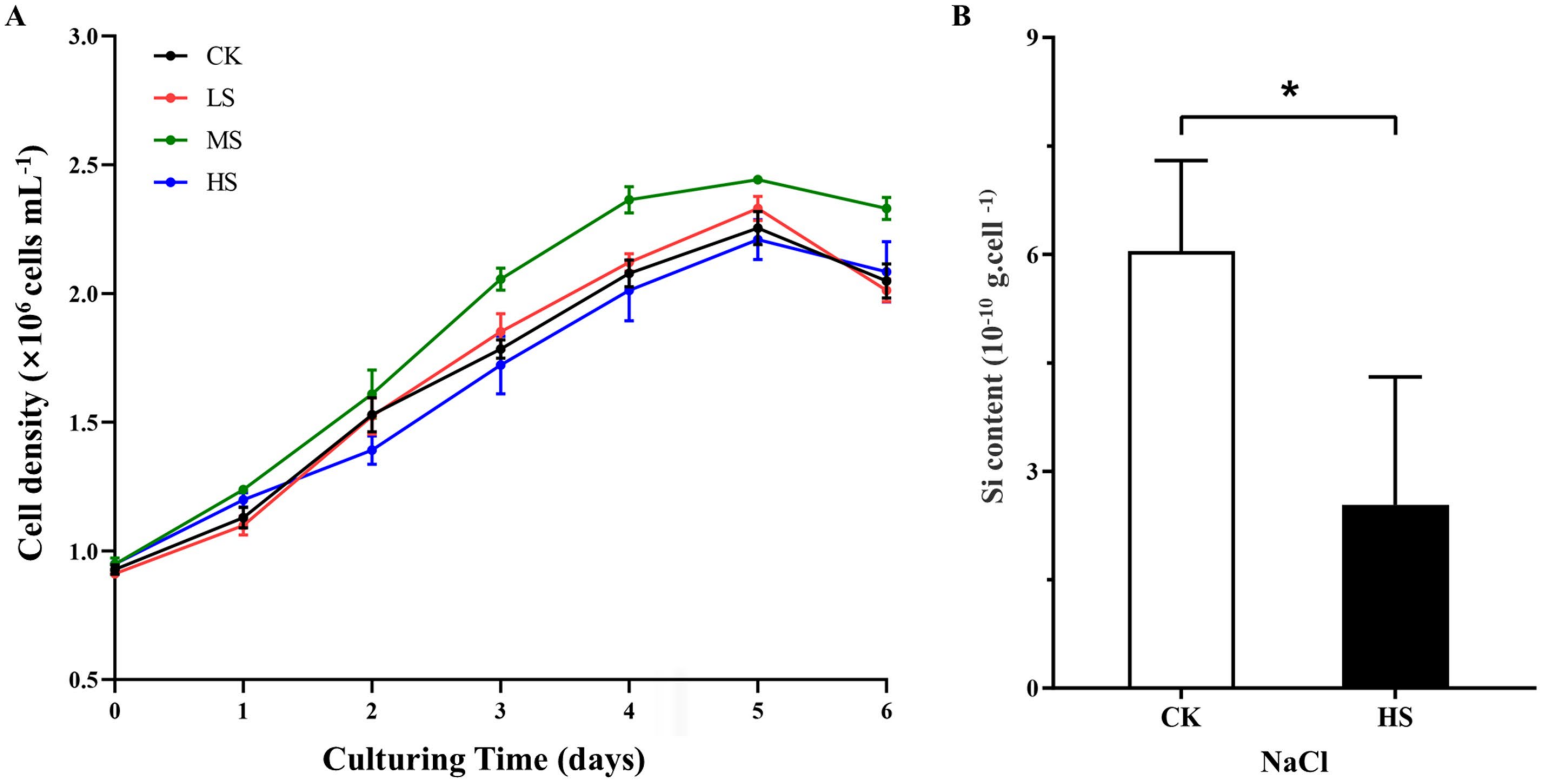
Physiological parameter variations of *Skeletonema subsalsum* grown in different salinities. (A) Cell density under four salinities. Data are means ± standard deviations (*n* = 3). Some standard deviations are not visible because they are shorter than the symbol size. (B) Silicon content in control (CK, salinity = 0) and higher salinity (HS, salinity = 12) treatments. The error bars represent the strand deviations (*n* = 3). The asterisk indicates significant differences between the CK and HS at *p* < 0.05.

The *S. subsalsum* cells were cultured in media with four different salinities. At each salinity, the cell density increased gradually and the cell growth entered the stationary phase after 4–5 days of culture. Although there were differences in cell density at different salinities, the cells maintained similar growth trends and cycles at all salinity levels (Figure 3A).

To better discuss the relationship between the expression level of DEGs and morphological changes, cells in the HS treatment with the most obvious morphological changes were selected and their silicon content was measured. Compared with the control (CK), the silicon content in cells decreased significantly in the HS treatment (Figure 3B).

### 3.3 Alterations of gene expression profiles in *Skeletonema subsalsum* under different salinity levels

RNA sequencing (RNA-Seq) was performed on samples collected from four salinity levels, with three independent biological replicates per treatment. Libraries were prepared and an average of 53,512,177 raw reads per sample were generated. Raw reads have been submitted to the National Center for Biotechnology (NCBI) with the accession number PRJNA1125160. This is the first gene expression profile of *S. subsalsum*. Results of quality control confirmed that the transcriptome sequencing results were of high quality and suitable for further analysis (Supplementary Tables S2, S3; Supplementary Figure S1A).

The assembled unigene sequences were then queried against six public databases to obtain annotations and the results are shown in Supplementary Figure S1B. In total, 59,933 genes (69.25%) were annotated in at least one database. Among them, most genes (51,167: 59.12%) presented sequences similar to known proteins in the eggNOG database.

### 3.4 Screening and analysis of DEGs

**Figure 4:**
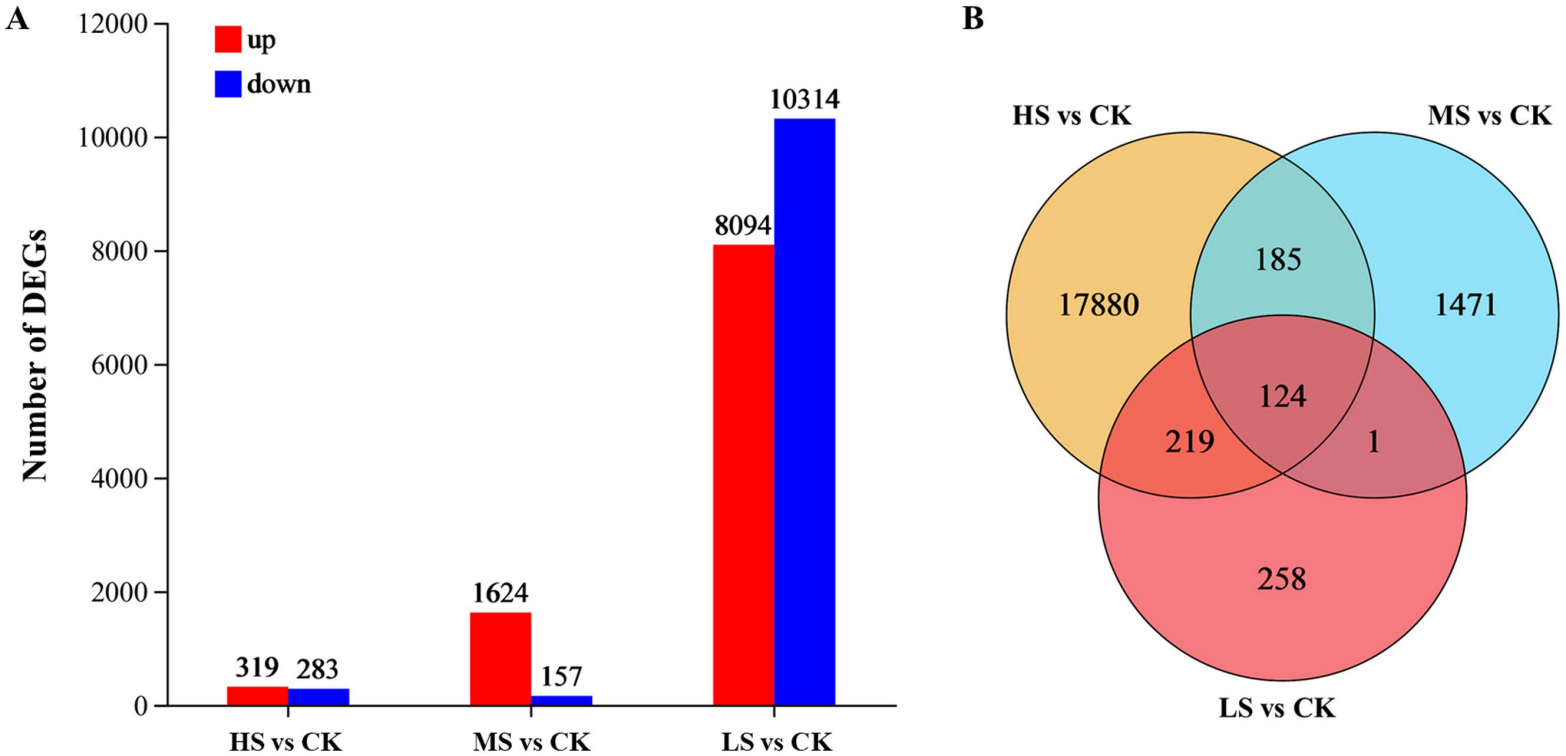
Comparison of all DEGs in *Skeletonema subsalsum* cells grown at various salinities. (A) Barplot of DEGs in LS, MS, and HS. Up-regulated and down-regulated DEGs are depicted in red and blue. (B) Overlap among the three treatments for all DEGs.

In this study, 20,138 DEGs in total were identified and analyzed (Figure 4). The results indicated that the LS treatment had the fewest DEGs (602), while the HS treatment had the most DEGs (18,408). In the MS treatment, 1,624 DEGs were upregulated, far greater than the number of downregulated DEGs (157) (Figure 4A). Compared with CK, 124 DEGs were shared among the three salinity levels, and 185 DEGs were shared between MS and HS, reflecting the significant differential expression of genes associated with the response to salinity stress. Of the unique DEGs, 258, 1,471, and 17,880 DEGs were specifically expressed in the LS, MS, and HS treatments, respectively (Figure 4B).

To clarify the transcriptional response, functional analysis was performed on all DEGs. A total of 15,154 DEGs were classified in 23 ontologies of the EggNOG database. Excluding 5,630 DEGs of unknown function, the number of DEGs classified in other ontologies is shown in Supplementary Figure S2. Interestingly, 386 and 224 DEGs were associated with “cell wall/membrane/envelope biogenesis” and “cytoskeleton,” respectively. In the GO database, 10,165 DEGs were annotated and classified into three main categories and 45 subcategories (Supplementary Figure S3A). Among the subcategories, “cellular process” and “metabolic process” were dominant in the biological process (BP) category, “cell part” was the largest subcategory in the cellular component (CC) category, and the subcategories of molecular function (MF) were mainly focused in “catalytic activity” and “binding.” KEGG annotation results showed that 6,247 DEGs were assigned to 22 pathways, and most of the DEGs were involved in “translation” (818 DEGs), “carbohydrate metabolism” (648 DEGs), “folding, sorting and degradation” (585 DEGs), “amino acid metabolism” (556 DEGs) and “transport and catabolism” (378 DEGs) (Supplementary Figure S3B). These dominant functions/pathways mentioned should be intimately linked to the salinity response of *S. subsalsum*.

**Figure 5:**
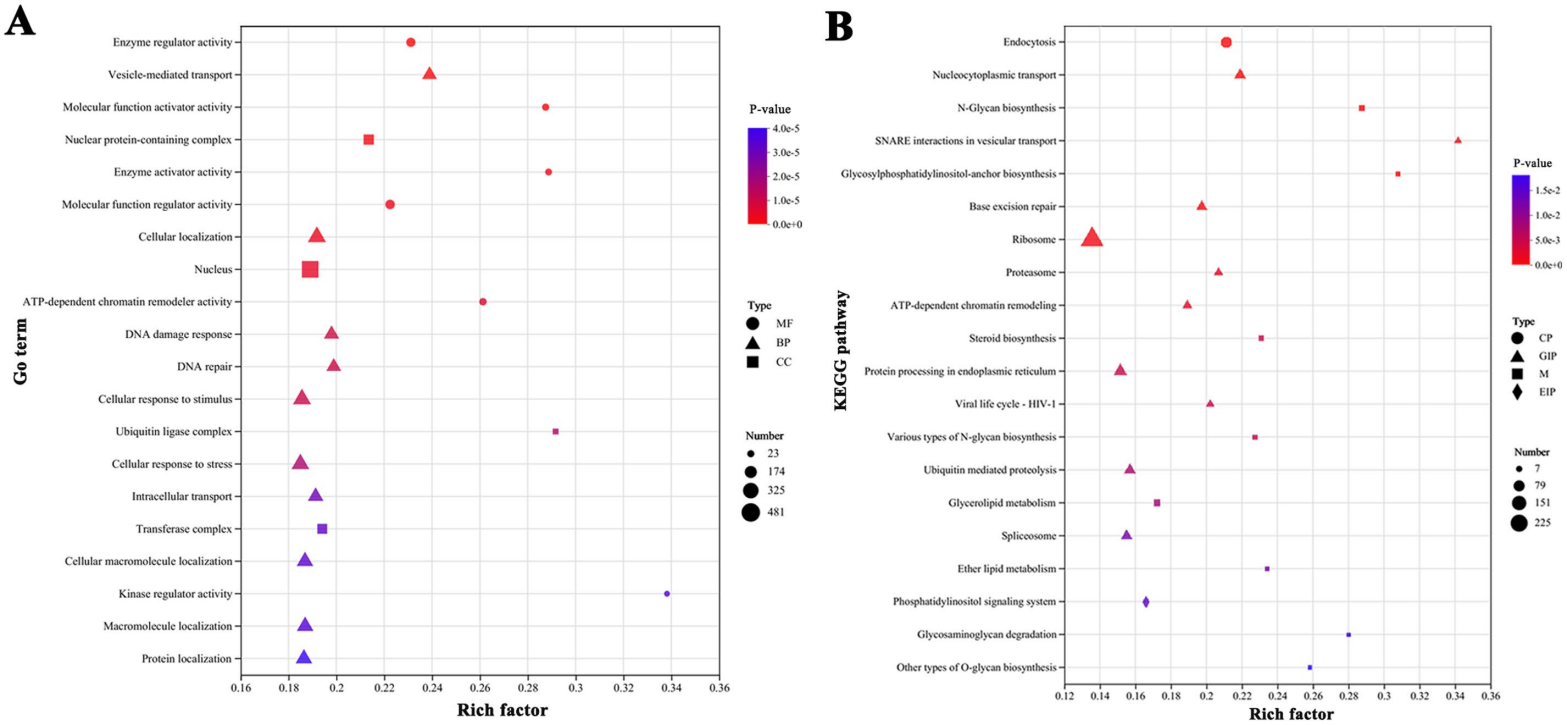
Functional enrichment analysis of screened DEGs showed significantly enriched terms or pathways. (A) GO enrichment analysis. (B) KEGG enrichment analysis. The Y-axis indicates the enriched GO terms or metabolic pathways, x-axis represents the ratio of the enriched genes number to the annotated. The dot size indicates the number of genes in the GO term or KEGG pathway, and the dot color corresponds to different *p*-value range. Only the top 20 enriched terms or pathways were showed using a corrected *p*-value ≤ 0.05 as the threshold.

Considering the characteristics of morphological changes, DEGs that exhibited the same variation trend in all treatment groups and were significantly expressed at all three salinity levels (THC), or were distinctly expressed in the HS and MS treatments (TWC), or were only prominently expressed in the HS treatment (ONS) were selected for further study. Ultimately, 9,061 DEGs that may be responsible for morphological changes were screened. Among these, 13, 142, and 4, 565 DEGs were upregulated in the THC, TWC, and ONS groups, respectively, while 11,143, and 4, 187 DEGs were downregulated in the THC, TWC, and ONS groups, respectively (Supplementary Figure S4). Enrichment analysis was conducted to describe the key biological functions of the screened DEGs. The results are presented in Figure 5. In the GO enrichment analysis, MF was the most enriched category and “kinase regulator activity” (23 DEGs) was the most enriched subcategory, followed by “ubiquitin ligase complex” (35 DEGs), “enzyme activator activity” (41 DEGs), and “molecular function activator activity” (46 DEGs) (Figure 5A). KEGG enrichment analysis indicated that the enriched terms mainly included “SNARE interactions in vesicular transport” (14 DEGs), “Glycosylphosphatidylinositol (GPI)-anchor biosynthesis” (16 DEGs) and “N-glycan biosynthesis” (25 DEGs) (Figure 5B). Importantly, “ubiquitin” and “vesicle transport” were enriched in both the GO and KEGG analyses. In addition, several pathways associated with glycan metabolism were enriched in KEGG analysis, including “O-glycan biosynthesis” and “various types of N-glycan biosynthesis” (15 DEGs). DEGs related to the above enrichment pathways are likely involved in cell wall formation. Discussing the changes in these DEGs is helpful to investigate the mechanism of morphological changes. The cytoskeleton is intimately linked to cell morphology, transport, and division. Although “cytoskeleton” was not enriched in the functional analysis, DEGs related to the cytoskeleton may still play an important role in the morphological changes.

### 3.5 Identification of DEGs related to morphological changes

Integrating the DEG functional analysis results and data from the literature showed that 231 of 9,061 DEGs were involved in cell wall formation (see Supplementary Table S4 for details). Their expression levels varied with salinity, resulting in significant morphological changes in *S. subsalsum*.

**Figure 6:**
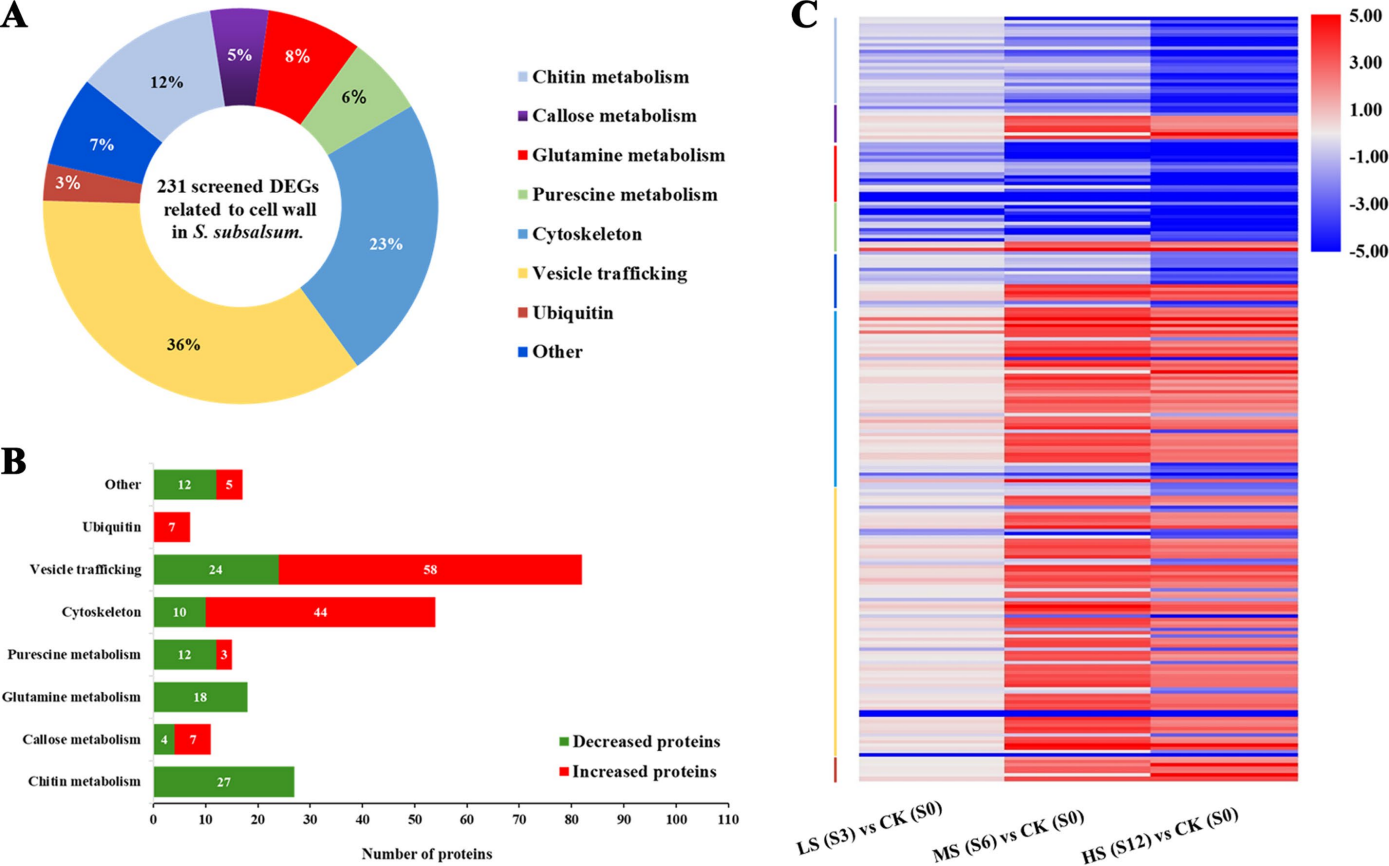
Analysis of differentially expressed genes (DEGs) associated with cell wall formation in *Skeletonema subsalsum* at various salinity levels. (A) Functional classifications were performed for 231 DEGs. (B) Functional categories of upregulated (red) and downregulated (green) salinity-related DEGs. (C) Heatmap of 231 DEGs and hierarchical clustering according to the functions of DEGs. The category colors correspond to the functional category colors shown in (A).

In total, 231 DEGs were classified into eight functional terms based on functional annotation. Among them, the most represented ontology was “vesicle trafficking” (36%), followed by “cytoskeleton” (23%) and “chitin metabolism” (12%) (Figure 6A). In all functional groups, four categories, namely “putrescine metabolism,” “glutamine (Gln) metabolism,” “chitin metabolism” and “other,” had more downregulated DEGs than upregulated DEGs (Figure 6B). The expression levels of DEGs in different functional groups under various salinity levels are shown in the heatmap (Figure 6C).

**Figure 7:**
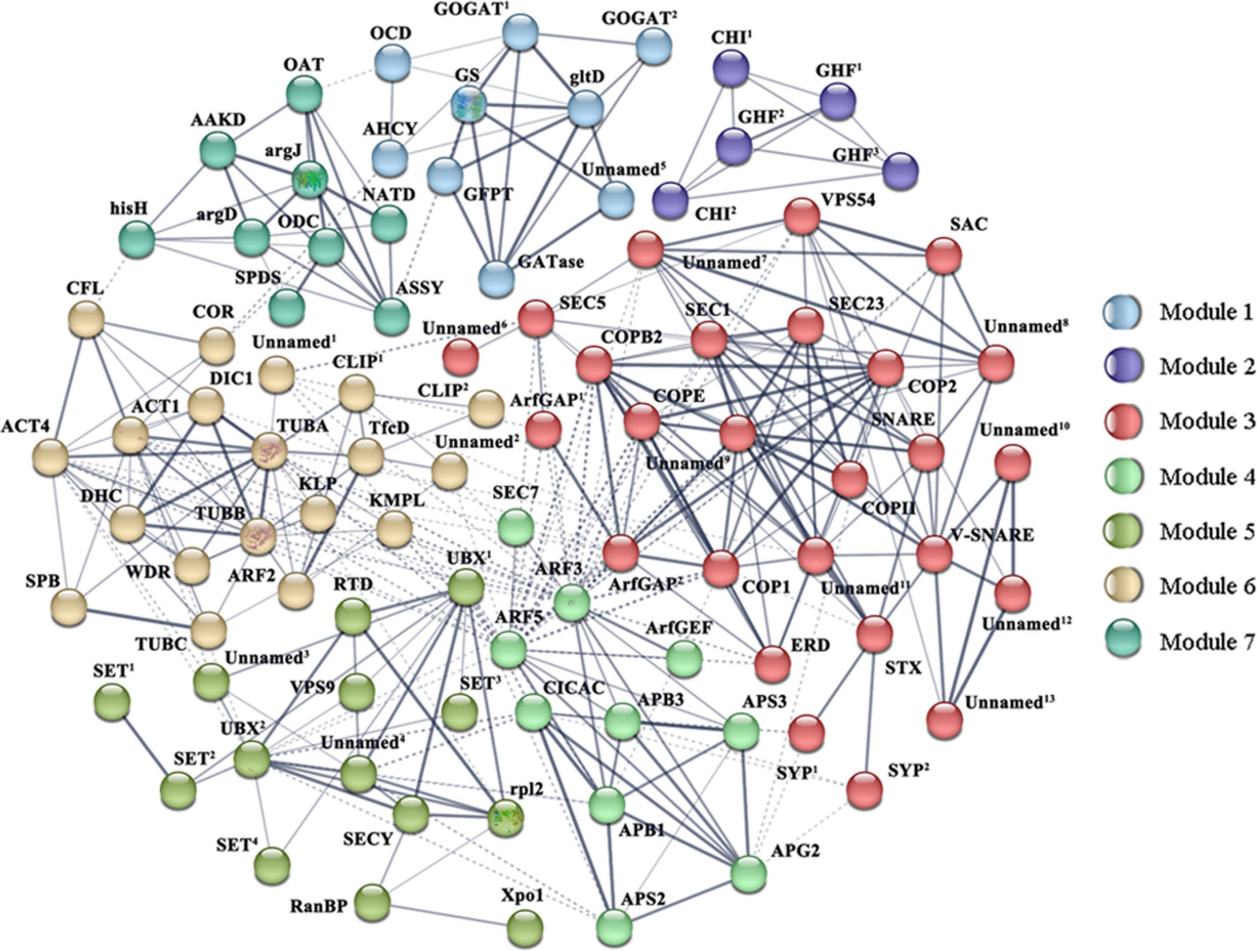
Protein–protein interaction (PPI) network in *Skeletonema subsalsum* revealed by STRING analysis. A total of 92 proteins related to cell wall formation are shown in the PPI network. Seven modules are presented in seven colors. Stronger associations are represented by thicker lines. Referring to this figure and Supplementary Table S5 for proteins abbreviations and corresponding DEGs.

Moreover, proteins encoded by 231 DEGs were used to construct a protein–protein interaction (PPI) network via STRING analysis based on homologous proteins in *Thalassiosira pseudonana*. Clusters function of STRING is used to mine the information of subnetworks. After removing the non-interacting proteins, 92 proteins were depicted in the PPI network, which may play more important roles in the cell wall formation, and divided into seven modules. Supplementary Table S5 shows proteins and the DEGs that codes for them. As shown in Figure 7, cytoskeleton proteins were mainly distributed in Module 6. These proteins interacted with the proteins in Modules 3 and 4, which were involved in vesicle transport, ubiquitin-related proteins in Module 5, and some of the proteins involved in putrescine metabolism in Modules 1 and 7. The diverse interactions validated that the cytoskeleton is indispensable in cell wall morphogenesis of diatoms. Proteins in Module 1 were linked with proteins in Module 7, these modules represented Glu and putrescine metabolism, respectively. In addition, ubiquitin-related proteins were highly connected with SET domain proteins and SECY in Module 5.

### 3.6 qRT-PCR verified the expression changes of DEGs at different salt concentrations

**Figure 8:**
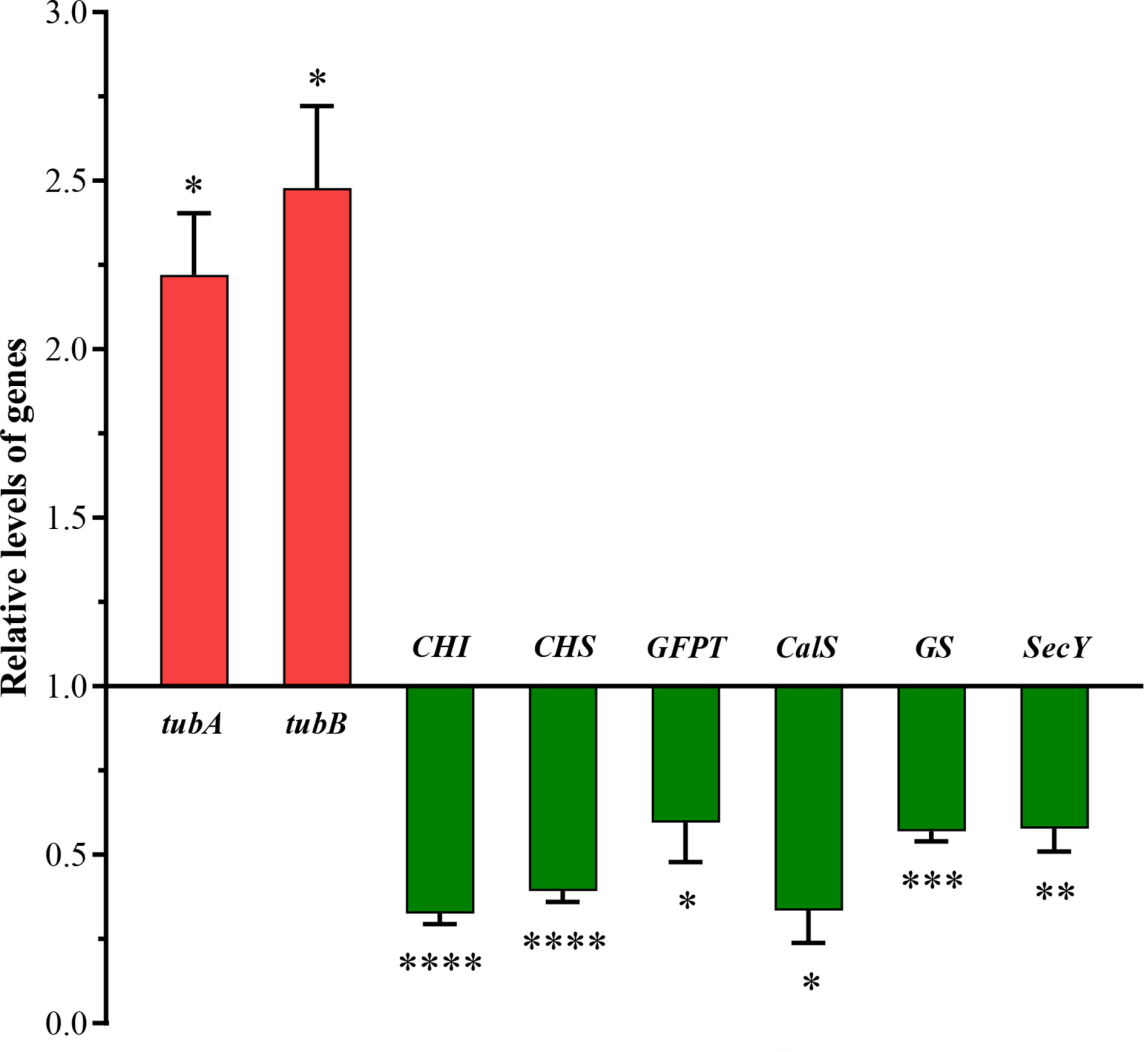
Quantitative RT-PCR (qRT-PCR) validations of DEGs in response to higher salinity. SSU rDNA was chosen as the internal reference gene. qRT-PCR relative expression levels were calculated using the 2^−ΔΔCt^ method presented on the left axis. The values are presented as means ± standard deviation (*n* = 3). *, **, ***, and **** indicate significant differences among CK and HS are *p* < 0.05, *p* < 0.01, *p* < 0.001, and *p* < 0.0001, respectively.

Based on the integrative analysis of the PPI and functional classifications, eight DEGs were selected for qRT-PCR assays to confirm the reliability of the transcriptomic analysis (Figure 8). Two of these DEGs were correlated with the cytoskeleton, including *tubA* and *tubB*, while the remaining DEGs were involved in silicon transport and deposition. Most of these DEGs were differentially expressed only in the HS treatment, therefore, the HS treatment was selected to perform qRT-PCR experiments. The results showed that most of the genes decreased with higher salinity, including *CHS*, *CHI*, *CalS*, *GFPT*, *GS*, and *SecY*. The expression trend of each DEG basically corresponded with its transcription level, indicating that the RNA-Seq results were accurate and reproducible.

## 4 Discussion

The salinity adaptation of diatoms has been a research hotspot in recent years. In the present study, we observed that salinity exerted significant effects on multiple structures in *S. subsalsum*. Consistent with previous reports, increases in salinity were associated with greater cell diameter and DBCs, decreased cell height, lengthened FPPs, and rounded frustules (Paasche et al., 1975; Hu et al., 2024). Particularly, it was also noted that the RPPs were lengthened and the pore size became larger with increasing salinity. Although the morphological changes of diatoms under salinity adaptation have been widely concerned, researches of the underlying molecular mechanisms are still in its infancy. In this study, the *de novo* transcriptome analysis was performed on *S. subsalsum* and 231 DEGs involved in cell wall development were screened to uncover the intrinsic causes of morphological changes. Similarly, Kamakura et al. (2024) proposed that salinity-induced morphogenetic changes in *P. laevis* are driven by the regulation of cytosolic Ca^2+^, Ca^2+^-dependent actin and frustule-related proteins. The molecular mechanism underlying salt-induced morphological changes is complex. It is necessary to conduct more comprehensive investigations of multiple diatom species that undergo morphological changes under salinity treatment.

### 4.1 Impact of salinity on silicon transport and deposition

The development of diatom cell walls has been studied for centuries (Rogall, 1939). To date, a variety of techniques have been used, including culture synchronization, material characterization, and molecular biology, among others, to interpret the processes of silicification (Kröger et al., 1999, 2000; Görlich et al., 2019) and to construct models (Hildebrand and Lerch, 2015). Currently, researchers generally agree that frustule formation includes at least six stages: (I) silicic acid is transported from the extracellular medium to the intracellular (Thamatrakoln and Hildebrand, 2008); (II) silica particles perhaps form in silica transport vesicles (STVs) (Liang et al., 2010); (III) STVs move to the silica deposition vesicle (SDV) membrane and release silica (Gordon et al., 2009); (IV) silica precipitates inside the SDV (Hazelaar et al., 2003) and a base layer forms; (V) the base layer thickens, accompanied by additional silicification; and (VI) the SDV is exocytosed (Pickett-Heaps et al., 1990; Haan et al., 2023). This suggests that both silicon transport and deposition are critical in cell wall morphogenesis. Focusing on the effects of salinity on the two processes will help researches uncover the mechanisms underlying morphological changes.

#### 4.1.1 Role of vesicles in silicon transport

The transport of substances between membranous organelles within cells is mainly accomplished by vesicles (Chen et al., 2019). In diatoms, vesicles are involved in biosilification at multiple levels, including SDV expansion, nanostructure shaping (Pickett-Heaps et al., 1990), and cell wall component transport (Du et al., 2014). In terms of component transport, STVs are suggested to be able to carry silicon and eventually fuse with the SDVs (Schmid and Schulz, 1979; Grachev et al., 2017), which is compatible with the presence of soluble silica pools in the form of silica nanoparticles (Kolbe and Brunner, 2022). Using Isobaric Tag for Relative Absolute Quantitation-based proteomic analysis, a previous work explored the silicon response mechanism of *T. pseudonana* and screened out five proteins, which were not only related to the vesicles but also were co-expressing proteins of SIT2. Du et al. (2014) claimed these proteins may be involved in silicon transport.

In the present study, we focused on whether DEGs encoding for these five proteins were present among the screened DEGs. Eventually, two DEGs that encoding for coatomer subunit beta 1 (COPB1) and coatomer subunit epsilon (COPE) were detected. The expression of the two DEGs was downregulated, consistent with the alterations of silicon content. Our findings implied that salinity may decrease the silicon transport within cells via interfering with vesicle transport. Undoubtedly, our speculation needs further confirmation, the intracellular localization of COPB1, COPE and silicon needs further studied. Remarkably, significantly downregulated *SecY* was found in all treatments, suggesting that *SecY* may associated with silicon transport and merits further investigation (Supplementary Table S4).

#### 4.1.2 Function of putrescine in silicon deposition

Putrescine is one of the most common polyamines, as well as a component of long-chain polyamines (LCPAs) (Kröger et al., 2000). Although LCPAs have only been studied *in vitro*, they have been confirmed to be abundant or‑ganic components of biosilica, which can accelerate the precipitation of silica (Kröger et al., 2002; Hildebrand et al., 2018). In this study, we noticed that many DEGs are involved in the metabolism of putrescine. The examination of the biochemical pathway showed that putrescine can be converted from Orn produced by arginine metabolism (Li et al., 2024). In our data, the expression of eight DEGs coding for multiple enzymes (acetylglutamate synthase, acetylglutamate kinase, acetylornithine aminotransferase, ornithine acetyltransferase and argininosuccinate synthase) in the synthetic pathway of arginine was decreased (Allen et al., 2011), accompanied by the downregulation of DEGs encoding ornithine decarboxylase (ODC), which catalyzes the formation of putrescine from Orn (Frigeri et al., 2006). The variation of DEGs indicated that salinity inhibited the synthesis of putrescine. Conversely, DEGs encoding for ornithine cyclodeaminase and D-ornithine 4,5-aminomutase, which can shuttle Orn away from putrescine synthesis, were dramatically upregulated (Supplementary Table S4).

Alternatively, putrescine is a precursor to the synthesis of spermine (Spm) and spermidine (Spd). Under the salinity treatments, the expression of the DEG (*Spm/Spd synthase*) related to Spm and Spd synthesis was stimulated (Wu et al., 2007), whereas genes encoding catabolic enzymes (*Polyamine oxidase*) were inhibited. These data showed that salinity reduced the intracellular accumulation of putrescine and increased the ratio of (Spm + Spd)/putrescine, which is correlated with membrane protection (Tiburcio et al., 1990; Besford et al., 1993). Similarly, Gong et al. (2014) has suggested that a higher (Spm + Spd)/putrescine ratio may be a crucial indicator in alkali stress responses.

An additional 12 salinity-inhibited DEGs encoding for the SET domain protein were identified in this study. This protein was first discovered in 2020 through the comprehensive comparative analysis of eight species of diatoms and is thought to regulate the methylation of substrate proteins and the functions of LCPAs (Nemoto et al., 2020). Overall, similar changes in SET-domain-related DEGs, putrescine metabolism, and silicon content imply that SET domain proteins may be involved in the methylation of putrescine, and putrescine formation and methylation may have a good correlation with silicon deposition. The key enzymes involved in putrescine metabolism are potential candidate genes for studying cell wall formation in diatom. This view is supported by Frigeri et al. (2006), who treated *T. pseudonana* with a specific inhibitor of ODC and examined thin and flexible cell walls. The findings of the present study indicated that salinity might diminish silicon deposition by inhibiting the metabolism and modification of putrescine, which finally resulted in the changes in silicon content and morphology.

#### 4.1.3 Function of chitin in silicon deposition

To probe the silicification process, researchers have investigated the composition of SDVs for decades (Shrestha et al., 2012; Kotzsch et al., 2017), leading to the seminal discovery that chitin is a major component of SDV (Shao et al., 2023). Chitin (Poly-N-acetyl-D-glucosamine), the second most abundant biopolymer on Earth, is a carbohydrate embedded in the cell walls of diatoms (Tesson et al., 2008; Cheng et al., 2021). Using methods including nuclear magnetic resonance spectroscopy and SEM, researchers have confirmed that chitin is coated with silica. Chitin acts independently of the rate of silicon polycondensation and is speculated to provide a scaffold for silica deposition (Waterkeyn and Bienfait, 1987; Brunner et al., 2009; Spinde et al., 2011).

Chitin metabolism is a reversible process. Wustmann et al. (2020) detected a chitin synthase in *T. pseudonana*, and found it was located specifically in a ring pattern in the region of the silicified girdle bands. Furthermore, Cheng et al. (2021) identified 243 chitin-related genes in *T. weissflogii* using PacBio sequencing. Correspondingly, 27 chitin-related DEGs encoding for six proteins were detected in the transcriptome analysis in this study (see Supplementary Table S4 for details). All DEGs were downregulated under salt treatment, and the downregulation level of most DEGs (24 DEGs) increased with increasing salinity. Transcriptomic results were supported by qRT-PCR results of *CHS* and *CHI*. This finding was similar to that of Downey et al. (2023), who shifted *Cyclotella cryptica* from seawater to freshwater and found that chitin metabolism was upregulated. On the whole, chitin and putrescine metabolism exhibited synergistic changes, providing genetic support for the theory that chitin acts as a scaffold for silicon deposition. In this study, salinity curbed the metabolism of chitin in *S. subsalsum*, indirectly affected silicon deposition, and thus changed the cell morphology.

#### 4.1.4 Function of ubiquitin in silicon deposition

Ubiquitin, common to all eukaryotic organisms, plays an irreplaceable role in the growth, development, and health of organisms (Doroodian and Hua, 2021). It is not only involved in the stability or degradation of proteins, but also in the cellular responses of plants to stress conditions (Liu et al., 2022). Particularly, biochemical and immunocytochemical localization studies on diatom, *Navicula pelliculosa,* have revealed that ubiquitin is present along the cell wall and inside nanopores, and may be involved in nanopore formation (Hazelaar et al., 2003).

In the present analysis, seven DEGs annotated as ubiquitin whose expression exhibited the same trend with morphological changes were screened. We detected that all of them exhibited increased expression under elevated salinity. The increase of ubiquitin may increase pores size and decrease cell silicification, which is in line with the morphological and physiological results of the present study. These results suggested that ubiquitin may related to the nanopore formation and negatively influences the degree of cell silicification. Previous reports had similar findings, with observations that cultured diatom species had smaller nanopores and thicker frustules under reduced salinity (Vrieling et al., 2000, 2007; Leterme et al., 2010). Collectively, salinity increased pore size by facilitating ubiquitin expression, bringing about a reduction in silicon content and a change in morphology.

### 4.2 Effect of salinity on the cytoskeleton

In diatoms, SDVs are surrounded by silicalemma (Reimann et al., 1966), which features silicalemma-spanning proteins that probably interact with the cytoskeleton, translating cytoskeletal assembly patterns into silica structures (Robinson and Sullivan, 1987). The centrality of the cytoskeleton in the PPI analysis also suggests that it is critical in cell wall formation.

#### 4.2.1 Regulation of microtubules in morphological changes

Microtubules (MTs) are a multifunctional part of a cell’s backbone and control cell architecture, division, and trafficking (Landskron et al., 2022). Using EM and MTs inhibitors, earlier work investigated and proposed multiple functions of MTs involved in valve formation in diatoms (Hildebrand and Lerch, 2015), including locating the MT coordination center, defining raphe, and expanding the SDVs. However, how the functions of MTs affect the size of SDVs remains ambiguous. This study took centric diatoms as the research object and attempted to put forward an opinion on this from the perspective of omics for the first time.

This study identified the enhanced expression of *tubA* and *tubB*, which are responsible for forming a heterodimer and are essential components of MTs (Miller et al., 2010) (Supplementary Table S4). Four DEGs related to tubulin folding cofactors, which contributes to tubulin folding and heterodimer formation (Zhou et al., 2023), were also upregulated. Furthermore, as another essential building block of MTs (Stathatos et al., 2023), two DEGs encoding *γ*-tubulin were increased under higher salinity. The expression changes of these DEGs suggested that salinity facilitated the assembly of MTs in *S. subsalsum*. Intriguingly, it was found that the enhancement of MT assembly coincided with an increase in frustule diameter, implying that MTs may decide the frustule diameter. Some previous immunofluorescence experiments conducted on centric diatoms supported this possibility. Meene and Pickett-Heaps (2002) visualized the MT structures of *Proboscia alata*, and observed MTs located at the edge of the SDV and thinnest part of valve. Furthermore, using fluorescence microscopy, Tesson and Hildebrand (2010) observed branching and radiating MT structures in *Coscinodiscus granii* that resembled superimposed line structures, and deduced that MTs strengthened the SDV.

Overall, the findings of this study suggest that MTs can positively regulate the expansion of SDVs, at least contributing to SDV extension in the x-axis direction, thereby controlling the frustule diameter of centric diatoms. Salinity enlarged the frustule diameter by promoting MT assembly in *S. subsalsum*.

Furthermore, this study monitored nine DEGs encoding for dynein and kinesin, which are MT-based motors that power intracellular cargo transport (Kendrick et al., 2019). The widespread downregulation of these DEGs under salt treatment was in accord with the changes in silicon-related vesicle transport, further demonstrating that salinity inhibited silicon transport in this study.

#### 4.2.2 Regulation of actin in morphological changes

Actin plays an important role in shaping the micro-scale structures of diatoms (Tesson and Hildebrand, 2010). In the past, researchers conducted inhibitor or immunofluorescence experiments to investigate the function of actin in cell wall formation (Schmid, 1980; Blank and Sullivan, 1983; Cohn et al., 1989). Significantly, Tesson and Hildebrand (2010) noticed that actin was located at the tips of the RPPs in *Triceratium dubium*, suggesting that actin is associated with the growth of RPPs.

Among the DEGs, the present work identified 22 DEGs encoding actin/actin related proteins, three DEGs (*Fimbrin*) related to actin crosslinking (Bretscher and Affiliations, 1981), and four DEGs (*Actin-related protein complex*) involved in actin nucleators (Goley and Welch, 2006) (Supplementary Table S4). The widespread upregulation of these DEGs was consistent with the increased lengths of FPPs and RPPs with higher salinity. The results imply that actin may be closely related to the lengths of FPPs and RPPs, which can be verified by further studies in combination with immunofluorescence or gene editing techniques.

## 5 Conclusion

In the present study, *S. subsalsum* was cultured at four salinity levels. Based on morphological observations and statistical analysis, this study found that with the increase of salinity, the cell diameter, pore diameter, DBCs, and protrusion lengths increased, while the cell height decreased. To reveal the intrinsic causes of salinity-induced morphological changes, physiological, transcriptomic, and qRT-PCR experiments were conducted on the cultured strains. Integrative analysis demonstrated that the morphological changes of *S. subsalsum* resulted from the synergy of multiple metabolic pathways regulated by multiple genes. Elevated salinity enhanced the expression of DEGs encoding MTs, actin, and ubiquitin, resulting in increased cell diameter, lengthened FPPs and RPPs, and enlarged pore size. In parallel, increased salinity inhibited chitin metabolism, vesicle transport, and putrescine metabolism and modification, thereby constricting silicon transport and deposition, ultimately leading to reduced silicification and a shortened cell height. Additionally, these findings provide new data on the cell wall formation mechanism from the perspective of salt tolerance. We proposed that genes encoding SECY, CHI, CHS, and key enzymes in putrescine metabolism are valuable candidate genes in cell wall formation. In the future, these genes can be examined in greater detail using methods such as gene editing.

## Data Availability

The datasets presented in this study can be found in online repositories. The names of the repository/repositories and accession number(s) can be found in the article/Supplementary material.
