# Supplementary material for: Transcriptomic analysis reveals the mechanism underlying salinity-induced morphological changes in Skeletonema subsalsum
Source: Front Microbiol. 2024 Oct 29;15:1476738. doi: 10.3389/fmicb.2024.1476738 (PMC11554505; doi:10.3389/fmicb.2024.1476738)
Supplement: Supplementary file 1 [file Data_Sheet_1.ZIP › Supplementary Materials/Supplementary Figures.docx]

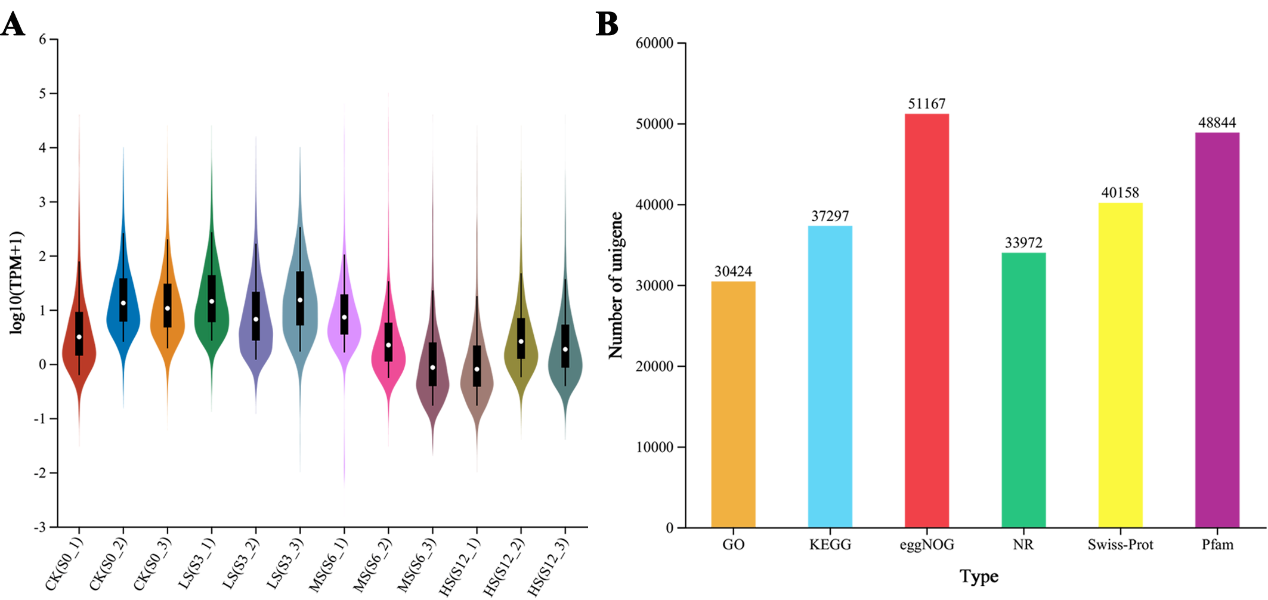


Supplementary Figure 1. Expression distribution and functional annotations of transcripts/unigenes. (A) TPM violin diagram present the expression distribution of transcripts in each sample. The enlarged part in the figure represents the region with the most concentrated expression of transcripts. (B) Results of unigene annotation against public database.


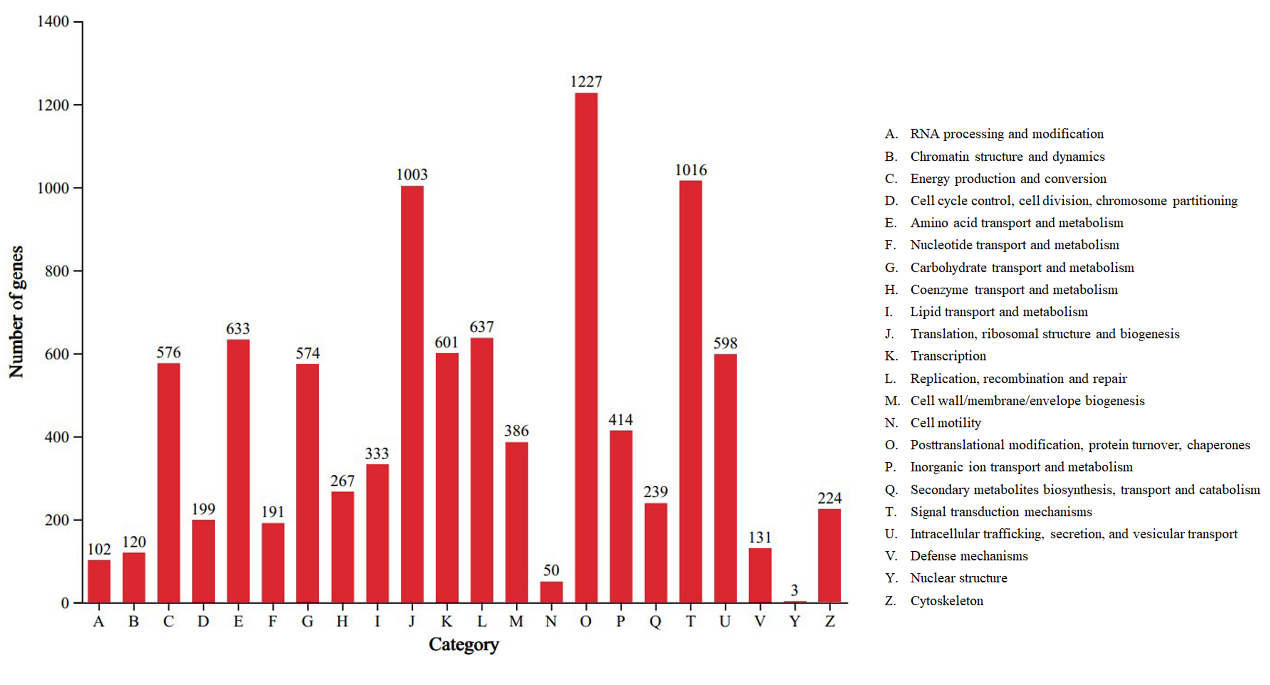


Supplementary Figure 2. EggNOG functional classification of all DEGs.


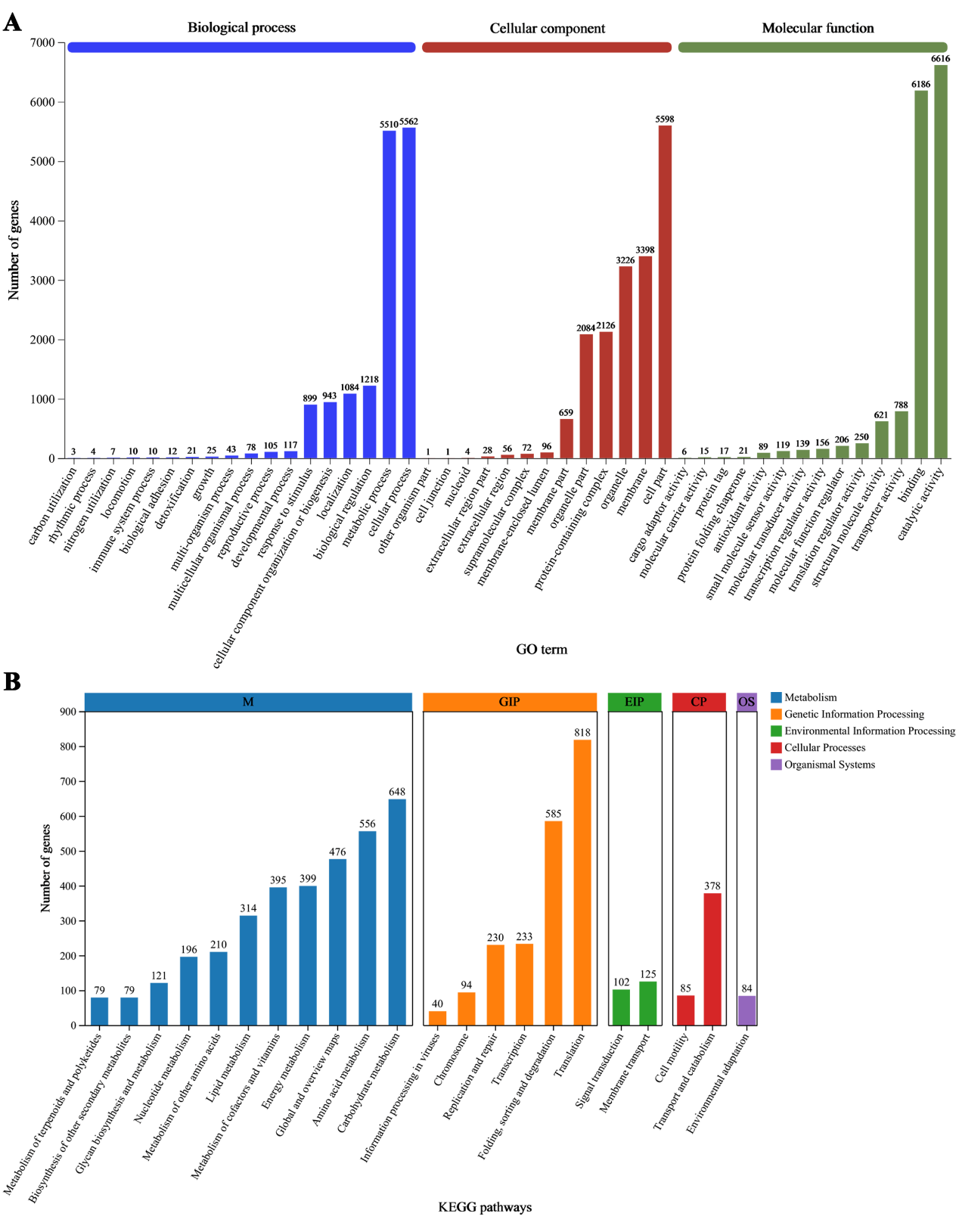
Supplementary Figure 3. GO and KEGG functional classification of all DEGs. (A) GO annotation. (B) KEGG annotation.


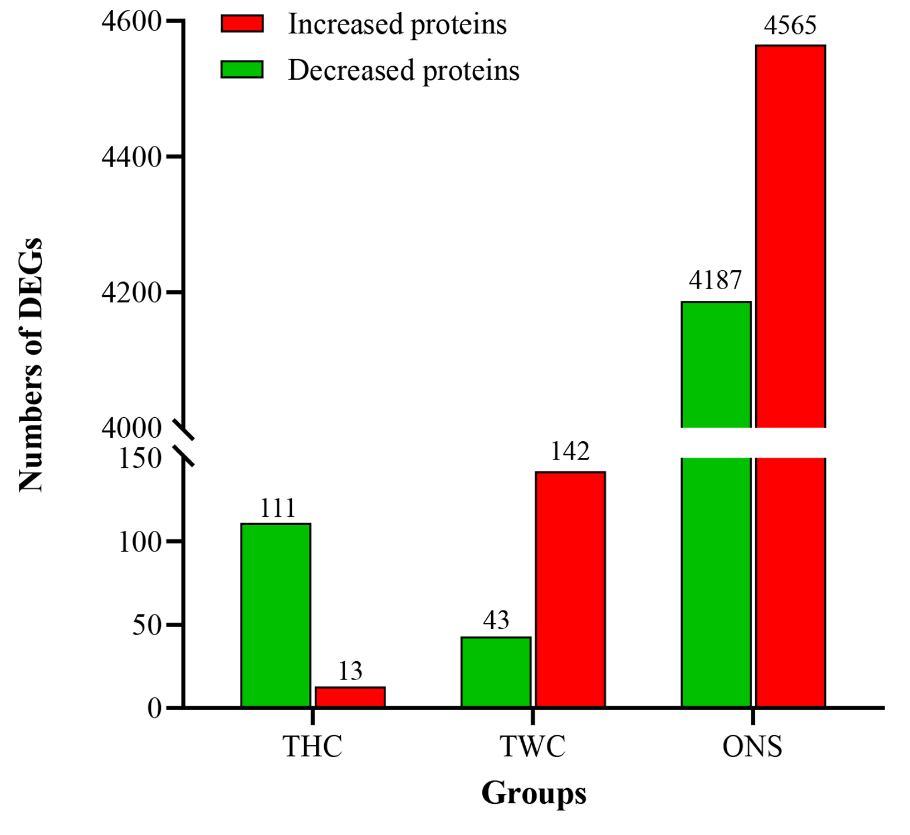
Supplementary Figure 4. Statistical analyses of DEGs changes at each group
