# Supplementary figures and images for: Transcriptomic analysis reveals the mechanism underlying salinity-induced morphological changes in Skeletonema subsalsum
Source: Front Microbiol. 2024 Oct 29;15:1476738. doi: 10.3389/fmicb.2024.1476738 (PMC11554505; doi:10.3389/fmicb.2024.1476738)

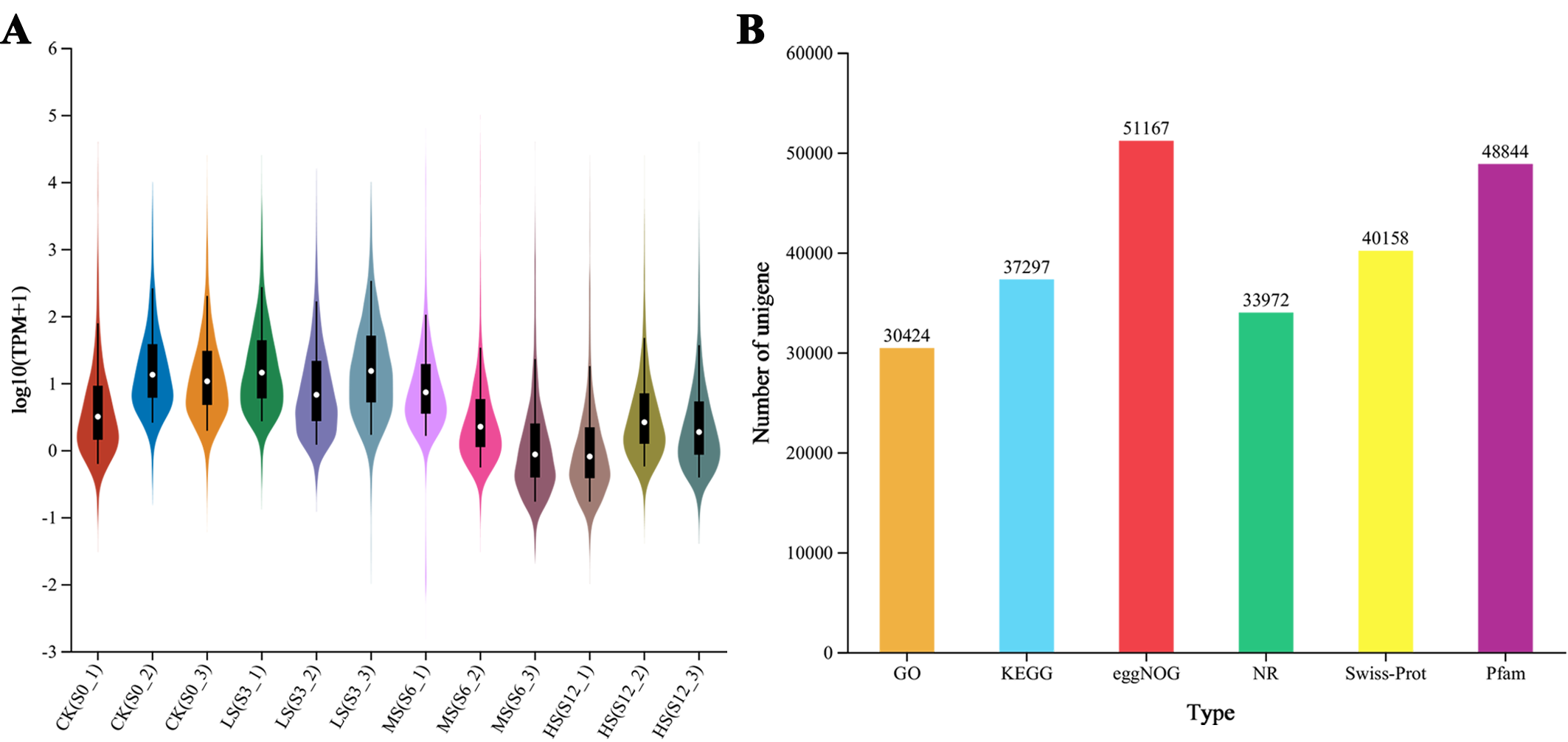

Supplement: Supplementary file 1 [file Data_Sheet_1.ZIP › Supplementary Materials/Supplementary Figure 1.tiff]

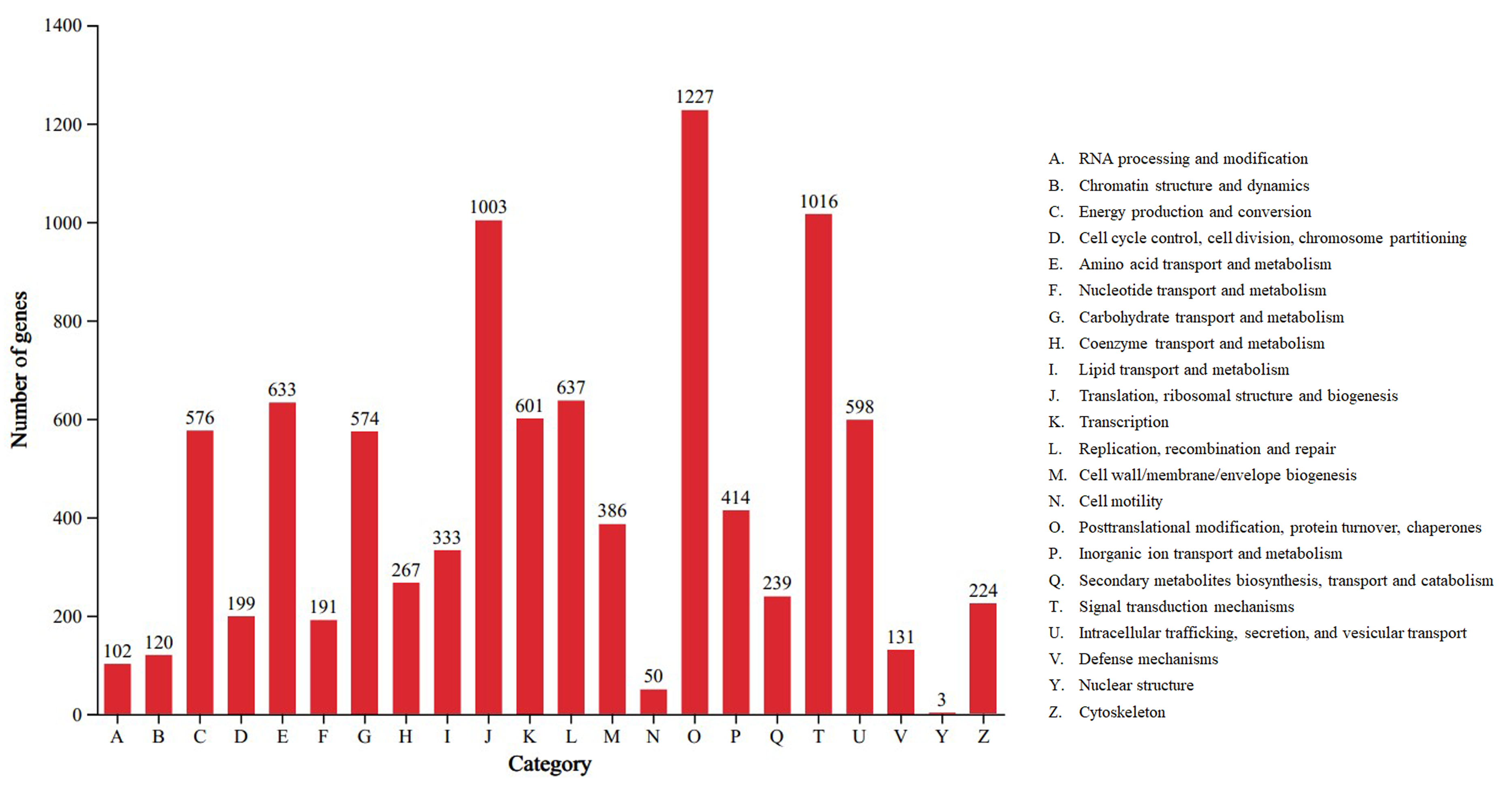

Supplement: Supplementary file 1 [file Data_Sheet_1.ZIP › Supplementary Materials/Supplementary Figure 2.tif]

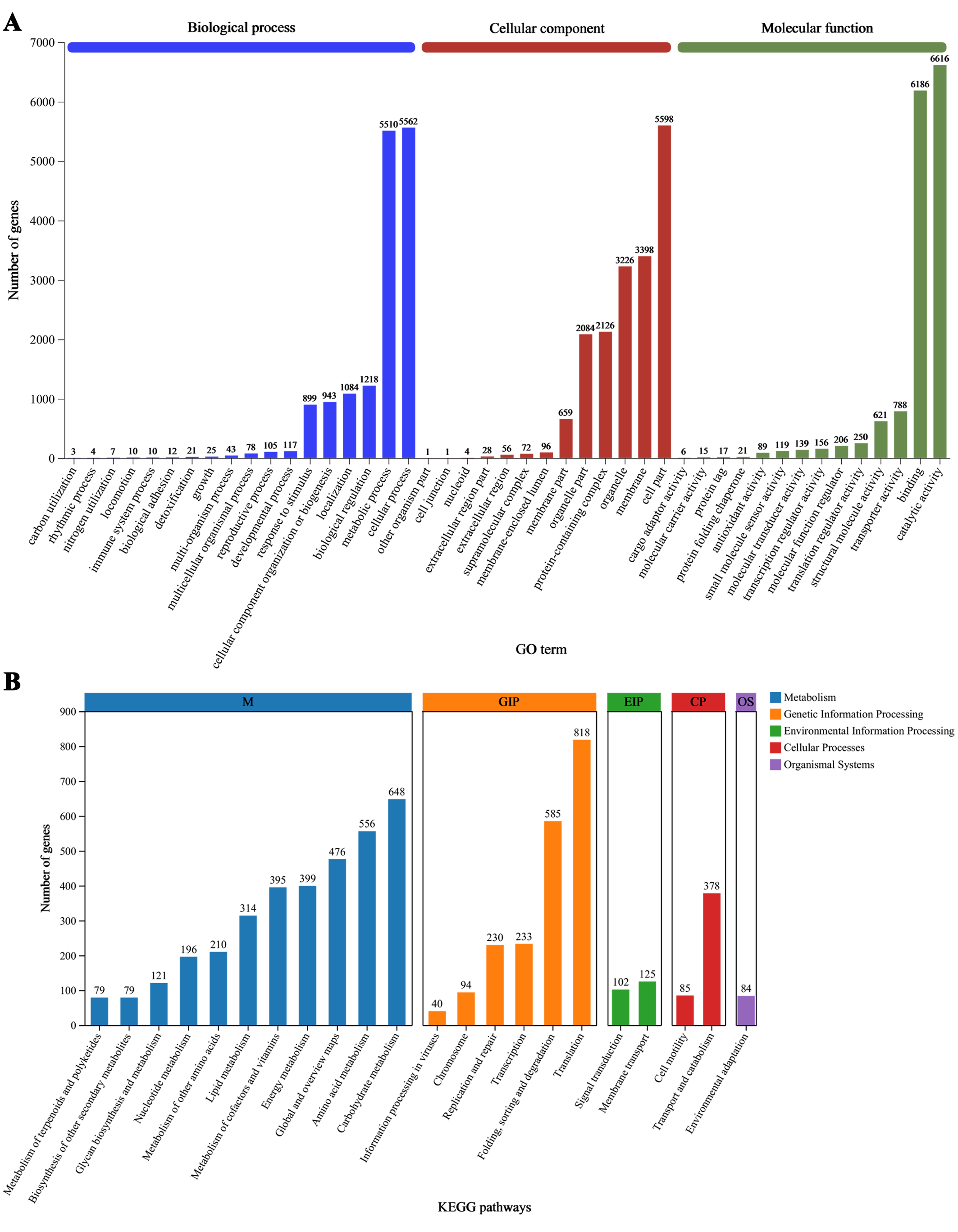

Supplement: Supplementary file 1 [file Data_Sheet_1.ZIP › Supplementary Materials/Supplementary Figure 3.tif]

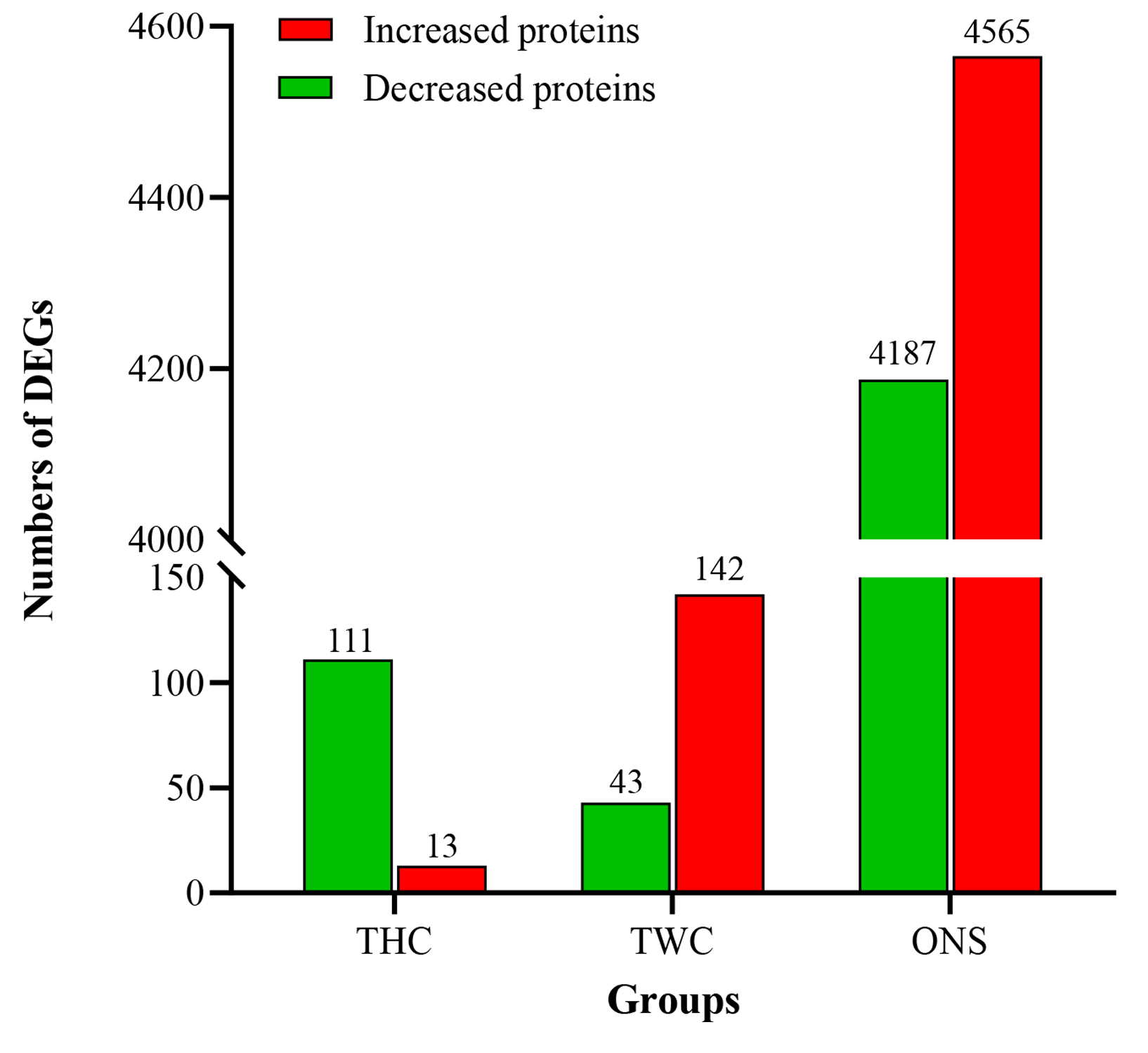

Supplement: Supplementary file 1 [file Data_Sheet_1.ZIP › Supplementary Materials/Supplementary Figure 4.tif]
